# Modified Checklist for Autism in Toddlers in a Neonatal High-Risk Population

**DOI:** 10.1001/jamanetworkopen.2026.3672

**Published:** 2026-03-27

**Authors:** Benjamin Lassebro, Matilda Morin, Weiyao Yin, Sven Sandin, Ulrika Ådén

**Affiliations:** 1 Department of Biomedical and Clinical Sciences, Linköping University, Linköping, Sweden; 2 Crown Princess Victoria Children’s Hospital, Linköping University Hospital, Linköping, Sweden; 3 Department of Medical Epidemiology and Biostatistics, Karolinska Institutet, Stockholm, Sweden; 4 Department of Psychiatry, Icahn School of Medicine at Mount Sinai, New York, New York; 5 Seaver Center for Autism Research and Treatment at Mount Sinai, New York, New York; 6 Department of Women’s and Children’s Health, Karolinska Institutet, Stockholm, Sweden; 7 Department of Neonatal Medicine, Karolinska University Hospital, Stockholm, Sweden

## Abstract

**Importance:**

Autism spectrum disorder (ASD) is a chronic neurodevelopmental condition with both genetic and environmental origins. Early detection is crucial for successful clinical intervention, improving future health, and adaptive functioning.

**Objective:**

To assess the diagnostic accuracy of the Modified Checklist for Autism in Toddlers (M-CHAT) in a neonatal high-risk population using subsequent clinical ASD diagnoses as the reference standard and to identify factors associated with the checklist’s accuracy within this group.

**Design, Setting, and Participants:**

This prospective, population-based cohort study identified children born in Sweden between January 1, 2013, and December 31, 2019, from the Swedish Neonatal Quality Register. Children were included if they were screened for autism using the checklist at corrected age 16 to 30 months. Follow-up for ASD in the National Patient Register extended from the day after inclusion or chronologic age 2 years, whichever occurred last, until December 31, 2022. Data were analyzed between June 6, 2024, and June 16, 2025.

**Exposure:**

Autism screening with the M-CHAT at 24-month neonatal follow-up.

**Main Outcomes and Measures:**

The primary outcome was the first recorded ASD diagnosis within the Swedish health care system among children categorized into the following neonatal high-risk groups: extremely preterm, small for gestational age, morphologic brain damage, neonatal encephalopathy, or other severe morbidity at birth. Diagnostic accuracy of the checklist was estimated using sensitivity, specificity, positive predictive value, and negative predictive value.

**Results:**

Among the 2178 children included in the study (median [IQR] corrected age at assessment, 26 [23-30] months; 1210 boys [55.6%]), 263 (12.1%) had positive screens. The overall sensitivity of the checklist was 62.4% (95% CI, 54.3%-69.7%); specificity, 91.2% (95% CI, 90.1%-92.4%), positive predictive value 31.4% (95% CI, 26.0%-37.1%), and negative predictive value 97.4% (95% CI, 96.7%-98.0%). Within the neonatal high-risk groups, children born extremely preterm had the highest proportion of positive screens and ASD diagnoses.

**Conclusions and Relevance:**

This cohort study found that in high-risk neonates, the M-CHAT had high specificity but moderate sensitivity when evaluated against later ASD diagnoses, highlighting the need for additional tools to improve detection.

## Key Points

**Question:**

What is the diagnostic accuracy of the Modified Checklist for Autism in Toddlers (M-CHAT) for identifying autism spectrum disorder (ASD) in a neonatal high-risk population?

**Findings:**

In this cohort study of 2178 high-risk neonates in Sweden, estimated sensitivity of the M-CHAT was 62.4%; specificity, 91.2%; positive predictive value, 31.4%; and negative predictive value, 97.4% when evaluated against a subsequent clinical ASD diagnosis.

**Meaning:**

The M-CHAT’s high specificity but moderate sensitivity for ASD highlights the need for additional tools to improve detection.

## Teaser

This cohort study assesses the diagnostic accuracy of the Modified Checklist for Autism in Toddlers in a neonatal high-risk population in Sweden and identifies factors associated with subsequent clinical diagnoses of autism spectrum disorder.

## Introduction

Autism spectrum disorder (ASD) is a chronic neurodevelopmental condition with genetic and environmental origins.^1^ Early detection is crucial for successful clinical intervention, improving future health, and adaptive functioning.^2,3^

The Modified Checklist for Autism in Toddlers (M-CHAT) is a screening tool designed for early detection of ASD in children aged 16 to 30 months.^4,5^ Diagnostic accuracy for the checklist varies across countries and study populations.^5,6^

Autism spectrum disorder is more common in children with neonatal complications, such as preterm birth, small for gestational age (SGA), and brain injuries, which often require neonatal intensive care unit treatment,^7,8,9^ compared with the general population.^10^ Therefore, the 23-item M-CHAT given at age 2 years is included in the Swedish neonatal follow-up program, a national initiative implemented in 2015 to provide structured identification and monitoring of long-term effects of preterm birth and neonatal illness.^11^ Children in this program, such as those born extremely preterm (<28 weeks’ gestation), often exhibit varying degrees of sensory, motor, and cognitive impairment, with a high prevalence of positive screens.^12^ These conditions may lead to misclassification when using the checklist to screen for autism.^13,14^ The usefulness of the checklist for this high-risk population has therefore been questioned. Additionally, boys are more likely than girls to screen positive on the checklist^15^ and have a higher prevalence of ASD diagnoses during childhood.^16^ Sex differences have been studied in primary care populations^15,17^ but have not yet been considered when evaluating the checklist’s performance in a high-risk population.

This study evaluated the sensitivity, specificity, positive predictive value (PPV), and negative predictive value (NPV) of the checklist as an ASD screening instrument in a neonatal high-risk population in Sweden, including 5 well-defined high-risk groups. A secondary objective was to identify factors associated with the checklist’s performance in this population.

## Methods

This prospective, population-based cohort study was approved by the Swedish Ethical Review Authority. Informed consent was not required according to Swedish law for register-based studies. The study was conducted in accordance with the Declaration of Helsinki^18^ and followed the Strengthening the Reporting of Observational Studies in Epidemiology (STROBE) reporting guideline.

### Data Sources

This study used data from Swedish national registers to estimate the diagnostic accuracy of the screening checklist for autism and subsequent ASD diagnosis in a neonatal high-risk population. The checklist’s results are reported to the Swedish Neonatal Quality Register (SNQ) as part of the follow-up program for high-risk neonates aged approximately 24 months.^19^ The SNQ data were linked to data from the National Patient Register (NPR),^20^ Medical Birth Register,^21^ Prescribed Drug Register,^22^ and Longitudinal Integrated Database for Health Insurance and Labor Market Studies.^23^ Linkage was enabled by the unique personal identity number assigned to all residents of Sweden.^24^

### Study Population

The study included all individuals born alive in Sweden between January 1, 2013, and December 31, 2019, with a checklist screening result at age 16 to 30 months, corrected for prematurity (calculated as chronologic age minus number of days born before 40 weeks’ gestation) recorded in the SNQ. Included children were from 1 of 5 defined neonatal high-risk groups: extremely preterm; SGA with a birth weight less than −3 SDs; morphologic brain damage (eg, intraventricular hemorrhage [IVH] grade 3-4 or stroke); neonatal encephalopathies (eg, hypoxic-ischemic encephalopathy [HIE] grade 2-3 and/or treated with therapeutic hypothermia); or other severe morbidity at birth (eg, critically impaired respiration or circulation). Classification of children into risk groups followed the specified reason for follow-up in SNQ, with the following exceptions: all children born at less than 28 weeks’ gestation were classified as extremely preterm for this study, and children for whom the reason for follow-up was not specified in SNQ were classified according to the first applicable high-risk group in the listed order (ie, extremely preterm, SGA, IVH recorded in SNQ, HIE recorded in SNQ). Children with an ASD diagnosis prior to the study inclusion date (date of the 24-month follow-up visit recorded in SNQ) were excluded to avoid potential bias in parental responses to the checklist, as prior knowledge of an ASD diagnosis could influence how parents interpret and report symptoms.

### Screening Instrument

The checklists exists in different versions, including the original 23-item version and the M-CHAT Revised With Follow-Up Interview.^4,5^ Within the Swedish neonatal follow-up program, the original 23-item checklist was administered in Swedish translation, with interpreter support for families with limited Swedish literacy.^25^ Among the 23 items, 6 critical items focusing on joint attention are considered the best discriminators of ASD.^4^ A positive screen is defined by failing 3 or more of any items or 2 or more critical items. Otherwise, the result is recorded as negative. A positive screen suggests possible ASD and is reviewed by a psychologist together with the parents to ensure that the parents understood the questions. After multidisciplinary testing, a team (including a psychologist, pediatrician, and physiotherapist) then decides whether further evaluation for ASD is warranted.^11^

### Outcome

The primary outcome was the first recorded ASD diagnosis assigned by specialists in the NPR from the day after study inclusion or chronologic age 2 years, whichever occurred last, until the end of follow-up (December 31, 2022). In Sweden, child health care is publicly funded and free of charge. Children with suspected ASD are referred to a specialist unit, which typically includes a psychologist and child psychiatrist, for assessment according to national recommendations using standardized tools such as the Autism Diagnostic Interview-Revised.^26^ We included the following *International Statistical Classification of Diseases, Tenth Revision* codes: F84.0, F84.1, F84.3, F84.5, F84.8, and F84.9. If the study inclusion date was missing, follow-up for ASD diagnoses started from corrected age 24 months.

### Other Covariates

Background characteristics, including gestational age at birth, child’s sex, and birth weight, were obtained from the Medical Birth Register. From SNQ, we obtained reason for neonatal follow-up; language spoken at home (a Scandinavian or non-Scandinavian language) at the time of screening; and diagnoses in the neonatal period, including bronchopulmonary dysplasia, necrotizing enterocolitis, persistent ductus arteriosus, IVH grade 3 to 4, HIE grade 2 to 3, and retinopathy of prematurity (detailed definitions of other covariates are provided in eTable 1 in Supplement 1). Due to national regulations and legal requirements in Sweden, data on race and ethnicity are neither requested nor collected.

### Statistical Analysis

The data analysis was performed between June 6, 2024, and June 16, 2025. Cumulative ASD incidence by age was summarized descriptively using inverse Kaplan-Meier curves (95% CIs) for children grouped by screening results and separately by sex. Children were followed up from corrected age 24 months or the date of neonatal follow-up until first emigration, death, first ASD diagnosis, or end of follow-up, whichever occurred first. We also summarized the median and 10th, 25th, 75th, and 90th percentiles of age at ASD diagnosis by screening result. In the primary analysis, we cross tabulated screening results (positive or negative) with ASD diagnosis at the end of follow-up and estimated M-CHAT’s accuracy for a later ASD diagnosis by calculating sensitivity, specificity, PPV, and NPV.^27^ These properties were estimated using the PROC FREQ procedure in SAS, version 9.4 (SENSPEC option) (SAS Institute Inc).^28^ Two-sided 95% CIs were constructed using a nonparametric bootstrap with 1000 resamples drawn with replacement from the analytic dataset. For each resample, estimates were recalculated and percentile intervals were defined by the 2.5th and 97.5th percentiles of the bootstrap distributions.^29^ We did not adjust for multiplicity of statistical tests.

#### Supplementary Analyses

In subgroup analyses, the checklist’s performance was estimated separately for each of the 5 neonatal high-risk groups. Performance was also estimated in subgroups by sex, for childhood autism (*International Statistical Classification of Diseases, Tenth Revision* code F84.0), and for ASD with co-occurring intellectual disability or attention-deficit/hyperactivity disorder (ADHD). The association of speaking a non-Scandinavian vs a Scandinavian language at home with the checklist’s performance was studied. Children with missing language information were excluded from this analysis.

To explore whether children with false-positive screens had received other developmental diagnoses during follow-up, we summarized the distribution of these diagnoses among children with positive screens and with and without an ASD diagnosis. Diagnoses included intellectual disability, ADHD, specific developmental disorders of speech and language or motor function, other or unspecified disorders of psychological development, or combinations thereof. To explore whether prescreening disabilities (motor, hearing, vision, neurosensory) were associated with increased false-positive screens, we tabulated ASD diagnoses at the end of follow-up among children with positive screens, stratified by disability type.

#### Sensitivity Analyses

The primary analysis was repeated with inclusion of only children born in 2017 or earlier (aged ≥5 years at the end of follow-up) to evaluate the potential influence of short follow-up for children born later. Next, we repeated the primary analysis while starting follow-up for ASD from age 3 years (in children born in 2018 and earlier) to avoid potential bias in parental responses to the checklist for children already under evaluation for ASD at the time of screening. The primary analysis was also repeated with inclusion of only children with a registered inclusion date, as children without a registered inclusion date might have been screened outside the recommended 16- to 30-month window. As a post hoc sensitivity analysis, we repeated the primary analysis by including children with an ASD diagnosis prior to the study inclusion date to assess the association of their exclusion with the outcomes. These children were originally excluded to minimize potential bias in parental responses to the checklist questionnaire.

## Results

### Population Characteristics

Among the 2178 children included in the study (median [IQR] corrected age at assessment, 26 [23-30] months; 1210 boys [55.6%] and 968 girls [44.4%]), 263 (12.1%) had positive screens, and 133 (6.1%) received an ASD diagnosis by the end of follow-up. The Figure shows the cumulative incidence of ASD by positive and negative screens. A total of 4705 children were registered for follow-up in SNQ between 2013 and 2019, of whom 2190 had an M-CHAT result at 16 to 30 months and were categorized into the predefined high-risk groups (eFigure 1 in Supplement 1). Twelve children were excluded because they died or emigrated before age 2 years or were diagnosed with ASD before study inclusion; 37 children were lost to follow-up after screening due to death or emigration.

**Figure.**
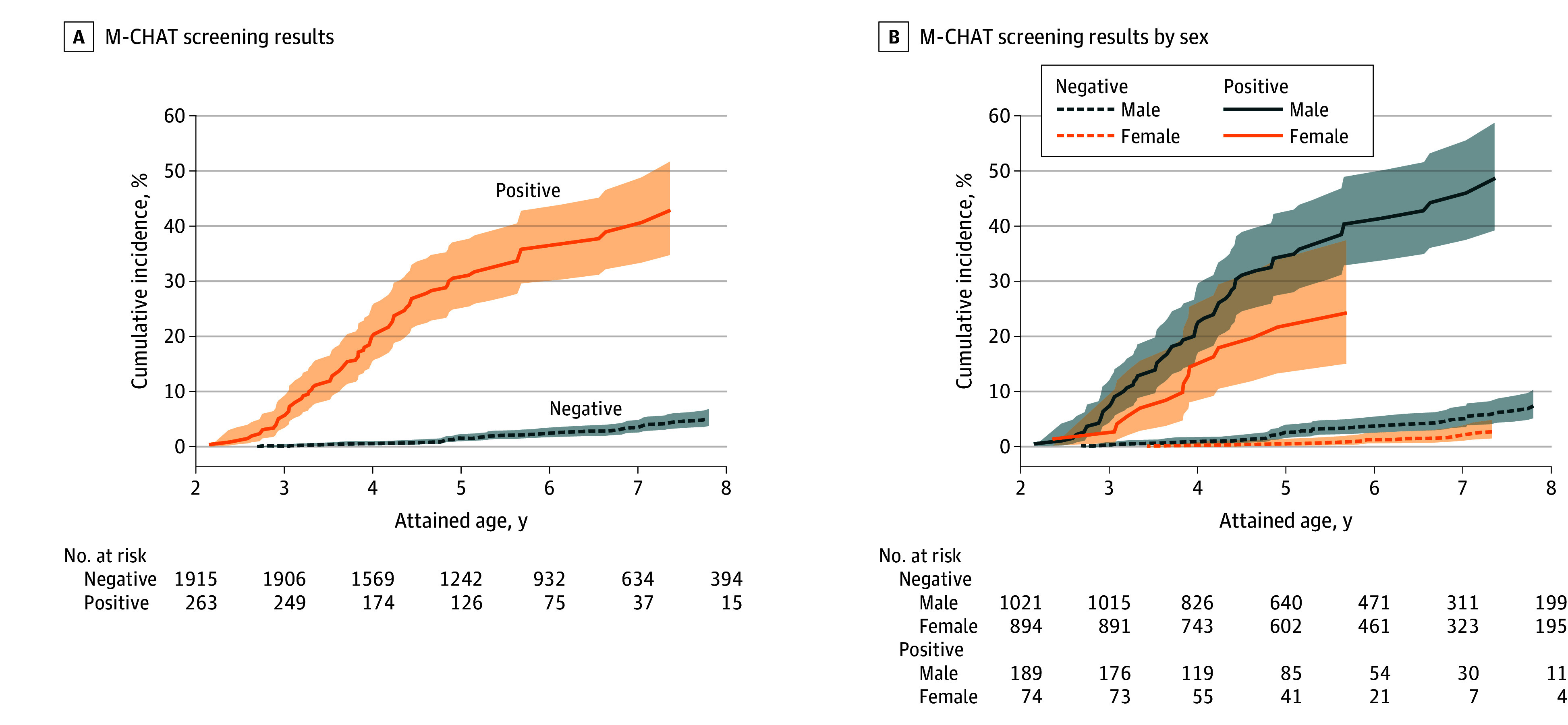
Inverse Kaplan-Meier Survival Curves for an Autism Spectrum Disorder (ASD) Diagnosis Between Age 2 and 9 Years A, The 10th percentile, median, and 90th percentile of age at ASD diagnosis in children with positive screens were 2.9, 3.9, and 5.6 years, respectively, and for children with negative screens, 3.3, 5.3, and 7.3 years, respectively. No events (ie, ASD diagnoses) occurred after age 7.8 years; thus, cumulative incidence was not estimable beyond this point. B, For female children with positive screens, the last event (ie, ASD diagnosis) occurred at age 5.7 years. Shading indicates the 95% CI.

Table 1 shows cohort characteristics overall and by screening result. Based on the descriptive statistics, the proportion of neonatal complications was higher among children with positive screens vs negative screens (bronchopulmonary dysplasia, 54.8% vs 30.4%; necrotizing enterocolitis, 9.5% vs 3.9%; persistent ductus arteriosus, 44.1% vs 24.5%; retinopathy of prematurity, 23.2% vs 8.9%), and parental education level was lower (mother education >12 years, 41.8% vs 55.4%; father education >12 years, 31.2% vs 41.6%). Among children with severe IVH (grade 3-4), 29 of 84 (34.5%) had positive screens (Table 1). Of the 84 children with severe IVH, 69 (82.1%) were born extremely preterm (eTable 2 in Supplement 1). In the overall cohort, extremely preterm birth was the largest neonatal high-risk category (870 children [39.9%]) (eTable 2 in Supplement 1). The proportion of children with positive screens was 18.3% in the extremely premature category, 12.6% in the morphologic brain damage category, and 7.2% in both the neonatal encephalopathy and SGA categories (Table 2).

**Table 1.** Cohort Characteristics Overall and With Positive and Negative Screening Results

Variable	Children, No. (%)		
	Total (N = 2178)	Screening results	
		Positive (n = 263)	Negative (n = 1915)
Child sex			
Female	968 (44.4)	74 (28.1)	894 (46.7)
Male	1210 (55.6)	189 (71.9)	1021 (53.3)
Birth weight, median (IQR), g^a^	1240 (570-4177)	859 (502-3825)	1307 (589-4189)
SGA with birth weight less than −3 SDs^a^	508 (23.8)	45 (17.4)	463 (24.6)
Gestational age, wk			
22-23	94 (4.3)	25 (9.5)	69 (3.6)
24-27	776 (35.6)	134 (51.0)	642 (33.5)
28-32	409 (18.8)	33 (12.5)	376 (19.6)
33-36	256 (11.8)	19 (7.2)	237 (12.4)
37-42	643 (29.5)	52 (19.8)	591 (30.9)
Bronchopulmonary dysplasia	727 (33.4)	144 (54.8)	583 (30.4)
Necrotizing enterocolitis	100 (4.6)	25 (9.5)	75 (3.9)
Persistent ductus arteriosus	586 (26.9)	116 (44.1)	470 (24.5)
IVH grade 3-4	84 (3.9)	29 (11.0)	55 (2.9)
Retinopathy of prematurity grade 3-5	231 (10.6)	61 (23.2)	170 (8.9)
HIE grade 2-3	193 (8.9)	20 (7.6)	173 (9.0)
Congenital abnormalities	878 (40.3)	139 (52.9)	739 (38.6)
Mother education, y			
≤9	246 (11.3)	49 (18.6)	197 (10.3)
10-12	714 (32.8)	98 (37.3)	616 (32.2)
>12	1170 (53.7)	110 (41.8)	1060 (55.4)
Missing	48 (2.2)	6 (2.3)	42 (2.2)
Father education, y			
≤9	284 (13.0)	52 (19.8)	232 (12.1)
10-12	893 (41.0)	107 (40.7)	786 (41.0)
>12	879 (40.4)	82 (31.2)	797 (41.6)
Missing	122 (5.6)	22 (8.4)	100 (5.2)
Maternal age, y			
<20	15 (0.7)	2 (0.8)	13 (0.7)
20-29	807 (37.1)	108 (41.1)	699 (36.5)
30-39	1203 (55.2)	132 (50.2)	1071 (55.9)
≥40	153 (7.0)	21 (8.0)	132 (6.9)
Language at home			
Scandinavian	1348 (61.9)	125 (47.5)	1223 (63.9)
Non-Scandinavian	503 (23.1)	93 (35.4)	410 (21.4)
Missing	327 (15.0)	45 (17.1)	282 (14.7)
Year of birth			
2013-2015	738 (33.9)	66 (25.1)	672 (35.1)
2016-2017	720 (33.1)	113 (43.0)	607 (31.7)
2018-2019	720 (33.1)	84 (31.9)	636 (33.2)
Corrected age at assessment median (IQR), mo^b^	26.0 (23.0-30.0)	26.0 (22.0-30.0)	26.0 (23.0-29.0)

Abbreviations: HIE, hypoxic ischemic encephalopathy; IVH, intraventricular hemorrhage; SGA, small for gestational age.

^a^ A total of 40 children (1.8%) were missing data on birth weight and SGA.

^b^ A total of 112 participants (5.1%) were missing data on age at assessment.

**Table 2.** Cross Tabulation of Screening Results and Clinically Assigned ASD Diagnoses During Follow-Up

Cohort and screening result	Children, No./total No. (%)	ASD diagnosis, No./total No. (%)
All children		
Positive	263/2178 (12.1)	83/263 (31.6)
Negative	1915/2178 (87.9)	50/1915 (2.6)
By sex		
Female		
Positive	74/968 (7.6)	15/74 (20.3)
Negative	894/968 (92.4)	12/894 (1.3)
Male		
Positive	189/1210 (15.6)	68/189 (36.0)
Negative	1021/1210 (84.4)	38/1021 (3.7)
By high-risk group		
Extremely preterm (<28 gestational wk)		
Positive	159/870 (18.3)	59/159 (37.1)
Negative	711/870 (81.7)	26/711 (3.7)
Small for gestational age		
Positive	30/416 (7.2)	8/30 (26.7)
Negative	386/416 (92.8)	7/386 (1.8)
Morphologic brain damage		
Positive	18/143 (12.6)	2/18 (11.1)
Negative	125/143 (87.4)	3/125 (2.4)
Neonatal encephalopathy		
Positive	30/419 (7.2)	7/30 (23.3)
Negative	389/419 (92.8)	8/389 (2.1)
Other severe morbidity		
Positive	26/330 (7.9)	7/26 (26.9)
Negative	304/330 (92.1)	6/304 (2.0)

Abbreviation: ASD, autism spectrum disorder.

### Diagnostic Accuracy of the M-CHAT

Overall checklist sensitivity was 62.4% (95% CI, 54.3%-69.7%), and specificity was 91.2% (95% CI, 90.1%-92.4%) (Table 3). By the end of follow-up, the PPV was 31.4% (95% CI, 26.0%-37.1%) for children with positive screens receiving an ASD diagnosis. In contrast, the NPV was 97.4% (95% CI, 96.7%-98.0%) for children with negative screens not receiving an ASD diagnosis.

**Table 3.** Diagnostic Accuracy of the Checklist for Detecting ASD

Population	Sensitivity, % (95% CI)	Specificity, % (95% CI)	PPV, % (95% CI)	NPV, % (95% CI)
Total population	62.4 (54.3-69.7)	91.2 (90.1-92.4)	31.4 (26.0-37.1)	97.4 (96.7-98.0)
Sex				
Female	55.6 (34.6-74.1)	93.8 (92.1-95.2)	20.0 (10.9-29.4)	98.7 (97.9-99.3)
Male	64.0 (54.4-73.2)	89.0 (87.3-90.9)	35.9 (28.8-42.7)	96.3 (95.0-97.5)
High-risk group				
Extremely preterm (<28 gestational wk)	69.4 (59.5-78.9)	87.3 (85.0-89.7)	36.9 (29.5-44.2)	96.4 (95.0-97.7)
Small for gestational age	53.3 (25.5-78.6)	94.6 (92.3-96.8)	25.9 (11.0-44.4)	98.2 (96.7-99.2)
Morphologic brain damage	40.0 (8.5-100.0)	89.1 (83.3-100.0)	13.3 (4.5-100.0)	97.6 (0.8-100.0)
Neonatal encephalopathy	46.2 (20.0-73.5)	94.3 (92.0-96.3)	22.6 (9.1-39.4)	98.0 (96.4-99.2)
Other severe morbidity	54.5 (27.3-83.3)	94.0 (91.4-96.3)	26.7 (10.5-45.2)	98.0 (96.4-99.4)

Abbreviations: ASD, autism spectrum disorder; NPV, negative predictive value; PPV, positive predictive value.

### Supplementary Analyses

Among the neonatal high-risk groups, the observed proportion of positive screens and ASD diagnoses was highest in the extremely preterm category (Table 2). However, the comparison of checklist performance between groups was underpowered due to small sample sizes, resulting in overlapping CIs (Table 3). The observed proportion of positive screens was 15.6% in boys and 7.6% in girls (Table 2). By the end of follow-up, 8.8% of boys and 2.9% of girls were diagnosed with ASD. Sensitivity of M-CHAT was 64.0% (95% CI, 54.4%-73.2%) for boys and 55.6% (95% CI, 34.6%-74.1%) for girls. Specificity was 89.0% (95% CI, 87.3%-90.9%) for boys and 93.8% (95% CI, 92.1%-95.2%) for girls (Table 3).

In supplementary analyses of M-CHAT performance in ASD subgroups, 128 of 133 children with ASD (96.2%) met criteria for childhood autism, 42 (31.6%) had ASD and a co-occurring intellectual disability, and 30 (22.6%) had ASD and ADHD (eTable 3 in Supplement 1). Sensitivity was estimated at 64.7% (95% CI, 56.5%-72.3%) for childhood autism, 71.1% (95% CI, 57.7%-83.5%) for ASD and a co-occurring intellectual disability, and 29.6% (95% CI, 13.5%-48.3%) for ASD and ADHD. Among children with a neurosensory or cognitive disability diagnosed before screening with the checklist, 38 of 113 (33.6%) had positive screens. In children without these conditions, 225 of 2065 (10.9%) had positive screens. The proportion of children later diagnosed with ASD was 31.6% in both groups (eTable 4 in Supplement 1).

A total of 125 of 1348 children (9.3%) with positive screens spoke a Scandinavian language at home, and 93 of 503 (18.5%) spoke a non-Scandinavian language. The checklist’s specificity was higher for children from Scandinavian-speaking households (93.3%; 95% CI, 91.9%-94.7%) compared with non-Scandinavian–speaking households (86.5%; 95% CI, 83.4%-89.5%). The PPV and NPV estimates did not show clear differences (eTable 5 in Supplement 1). A total of 327 children with missing home language information were excluded from this analysis.

A majority of children with positive screens who were diagnosed with ASD also received other developmental diagnoses during follow-up, including intellectual disability and other psychological development disorders (eFigure 2 in Supplement 1). Among children with positive screens who were not diagnosed with ASD, 115 of 180 (63.9%) did not receive any developmental diagnosis during follow-up.

Sensitivity analyses under alternative assumptions are presented in eTable 6 in Supplement 1. Estimates for sensitivity, specificity, PPV, and NPV varied slightly across analyses but did not change the overall interpretation of checklist’s performance.

## Discussion

This cohort study of 2178 children in Sweden with neonatal complications and an elevated risk of ASD found that the M-CHAT administered at corrected age 24 months had a high specificity (91.2%) but moderate sensitivity (62.4%) for an ASD diagnosis at age 2 to 9 years. While effectively excluding an ASD diagnosis, the checklist’s ability to identify ASD was more limited in this high-risk population. Sensitivity of 70% to 80% and specificity greater than 80% are considered desirable for screening tests^30,31^; therefore, use of the checklist in high-risk neonates should be combined with complementary measures and clinical assessment to avoid missing children who could be diagnosed with ASD.

The observed sensitivity was lower than that found in a recent meta-analysis of M-CHAT performance, in which a pooled sensitivity of 83% in low- and high-risk samples was reported.^6^ Methodological differences complicate comparisons. For example, sensitivity may be inflated if only children with positive screens undergo further evaluation, preventing identification of false-negative findings and resulting in an artificial sensitivity of 100%.^32,33^ When the meta-analysis was restricted to studies using confirmation strategies similar to ours (registers or medical record review), sensitivity dropped to 57% in primary care populations,^6^ comparable with our findings. Specificity of the checklist may vary by population ASD risk. Although theoretically independent of prevalence, co-occurring developmental conditions in high-risk groups may increase false-positive results. In our cohort, however, specificity remained high at 91.2%, despite the elevated risk profile. Another methodological difference is that we used the original 23-item checklist, whereas many studies have used the revised checklist with follow-up interview or implemented repeated screening, both associated with improved performance.^5,34^

This study is the first, to our knowledge, to evaluate the checklist’s diagnostic accuracy against subsequent clinical ASD diagnoses in a large neonatal intensive care unit–treated cohort. Various aspects of the checklist’s performance have been assessed in preterm populations, with varying results. In our cohort, 18.3% of extremely preterm children screened positive, similar to the 21% reported by Kuban et al.^35^ Moore et al^12^ reported a higher rate (41%), which may have been due to inclusion of children born at less than 27 weeks’ gestation (as lower gestational age is associated with increased ASD risk^36^) and because screening was performed at chronologic rather than corrected age 24 months (as younger screening age is associated with higher rates of M-CHAT positive screens^37^). Among children with severe IVH (grade 3-4), Shehzad et al^9^ reported 43% positive screens using the checklist, while in our cohort, 29 of 84 children (34.5%) with IVH screened positive, most of whom were also born extremely preterm.

Sex and language spoken at home influenced the checklist’s specificity in this study. Specificity was higher for girls than for boys. This finding may align with studies indicating a higher genetic and phenotypic variance in boys.^38,39^ To our knowledge, only a few studies have examined the checklist’s differences by sex, reporting similar sensitivity and specificity in low-risk children but higher PPVs in boys,^15,17^ possibly due to a higher ASD prevalence in boys.^16,40^ Higher specificity among children from families who spoke a Scandinavian language at home suggests the need for improved screening tools for immigrant populations.

The checklist showed lower sensitivity (29.6%) for children with co-occurring ASD and ADHD than for those with ASD alone (62.4%) or ASD with an intellectual disability (71.1%) (eTable 3 in Supplement 1). This finding may indicate a reduced ability for the checklist to identify ASD when inattention and hyperactivity are present, potentially leading to alternative interpretations of developmental concerns. A preexisting ADHD diagnosis has been shown to delay ASD diagnosis in children, particularly girls.^41^

Parent-reported disabilities at screening, especially substantial motor, hearing, and vision impairments, increase the risk of a positive screen.^12^ In our cohort, positive screens were more common among children with at least 1 cognitive or neurosensory disability diagnosed before screening (33.6% vs 10.9% in children without such disabilities) (eTable 4 in Supplement 1). However, the proportion children with positive screens later diagnosed with ASD was identical in those with and without a cognitive or neurosensory disability (31.6%), arguing against an increase in false-positive test results and suggesting similar PPVs in these subgroups. The low number of children with disabilities precluded further analyses.

### Strengths and Limitations

A strength of our study was the population-based design within a publicly funded health system providing comprehensive developmental follow-up (≥8 times from age 0 to 5 years for all children),^42^ minimizing selection biases. Other strengths included the substantial sample size and prospectively collected clinical ASD diagnoses. These features provided for a robust evaluation of the checklist’s performance in neonatal high-risk populations across diverse etiologies and offered clinically relevant insights for interpreting screening results.

The study also had some limitations. The youngest participants were aged 3 years at follow-up (median, 6 years), and some may have received an ASD and/or other developmental diagnosis later, introducing potential misclassification. We addressed misclassification in a sensitivity analysis restricted to children aged 5 years or older, which showed checklist sensitivity and specificity comparable to the primary analysis. Despite the large overall sample size, statistical power was limited for detecting differences when studying specific neonatal high-risk subgroups. Finally, the SNQ included only the final screening result (positive or negative), which prevented any item-level analyses.

## Conclusions

This cohort study is the first, to our knowledge, to evaluate the diagnostic accuracy of the M-CHAT compared with subsequent clinical diagnoses of ASD in a large neonatal high-risk population born extremely preterm, SGA, or with severe morbidity at the national level in Sweden. Our findings suggest that the checklist has high specificity but moderate sensitivity in this setting, indicating the need for additional tools to improve detection.

## Notes

Supplement 1. **eTable 1.** Definition of Covariates/Diagnoses **eTable 2.** Participant Characteristics by Neonatal High-Risk Group **eTable 3.** Performance of the Modified Checklist for Autism in Toddlers for a Diagnosis of Childhood Autism, ASD+ID, and ASD+ADHD **eTable 4.** Disabilities Present at Age 2 Years, Positive Modified Checklist for Autism in Toddlers Screens, ASD Diagnoses at the End of Follow-Up **eTable 5.** Association of Having Another Language Than a Scandinavian Language Spoken at Home With Performance of Modified Checklist for Autism in Toddlers Screening to Estimate an ASD Diagnosis **eTable 6.** Sensitivity Analyses **eFigure 1.** Flowchart of Study Population **eFigure 2.** Proportion of Other Developmental Disorders Among Individuals Who Screened Positive on the Modified Checklist for Autism in Toddlers, by ASD Status

Supplement 2.
Data Sharing Statement
