# Supplementary material for: Modified Checklist for Autism in Toddlers in a Neonatal High-Risk Population
Source: JAMA Netw Open. 2026 Mar 27;9(3):e263672. doi: 10.1001/jamanetworkopen.2026.3672 (PMC13032150; doi:10.1001/jamanetworkopen.2026.3672)
Supplement: Supplement 1. — eTable 1. Definition of Covariates/Diagnoses eTable 2. Participant Characteristics by Neonatal High-Risk Group eTable 3. Performance of the Modified Checklist for Autism in Toddlers for a Diagnosis of Childhood Autism, ASD+ID, and ASD+ADHD eTable 4. Disabilities Present at Age 2 Years, Positive Modified Checklist for Autism in Toddlers Screens, ASD Diagnoses at the End of Follow-Up eTable 5. Association of Having Another Language Than a Scandinavian Language Spoken at Home With Performance of Modified Checklist for Autism in Toddlers Screening to Estimate an ASD Diagnosis eTable 6. Sensitivity Analyses eFigure 1. Flowchart of Study Population eFigure 2. Proportion of Other Developmental Disorders Among Individuals Who Screened Positive on the Modified Checklist for Autism in Toddlers, by ASD Status [file jamanetwopen-e263672-s001.pdf]

Supplemental Online Content

Lassebro B, Morin M, Yin W, Sandin S, Ådén U. Modified Checklist for Autism in Toddlers in a neonatal high-risk population. *JAMA Netw Open*. 2026;9(3):e263672.
doi:10.1001/jamanetworkopen.2026.3672

eTable 1. Definition of Covariates/Diagnoses

eTable 2. Participant Characteristics by Neonatal High-Risk Group

eTable 3. Performance of the Modified Checklist for Autism in Toddlers for a Diagnosis of Childhood Autism, ASD+ID, and ASD+ADHD

eTable 4. Disabilities Present at Age 2 Years, Positive Modified Checklist for Autism in Toddlers Screens, ASD Diagnoses at the End of Follow-Up

eTable 5. Association of Having Another Language Than a Scandinavian Language Spoken at Home With Performance of the Modified Checklist for Autism in Toddlers Screening to Estimate an ASD Diagnosis

eTable 6. Sensitivity Analyses

eFigure 1. Flowchart of Study Population

eFigure 2. Proportion of Other Developmental Disorders Among Individuals Who Screened Positive on the Modified Checklist for Autism in Toddlers, by ASD Status

This supplemental material has been provided by the authors to give readers additional information about their work.

© 2026 Lassebro B et al. *JAMA Network Open*.

eTable 1. Definition of Covariates/Diagnoses

Variable	Data source	Definition
ASD	NPR	ICD-10: F84.0, F84.1, F84.3, F84.5, F84.8, F84.9
Childhood autism	NPR	ICD-10: F84.0
ASD with co-occurring ADHD	NPR or PDR	ASD diagnosis plus ICD-10: F90 or filled prescription of ATC N06BA04 (methylphenidate), N06BA09 (atomoxetine), N06BA12 (lisdexamfetamine), C02AC02 (guanfacin), N06BA02 (dexamfetamine) anytime during follow-up
ASD with co-occurring ID	NPR	ASD diagnosis plus ICD-10: F70–F79 anytime during follow-up
Congenital abnormalities	NPR	Any diagnosis of ICD-10 chapter Q before age 2
Disabilities at age 2		
Cognitive disability	NPR	ICD-10: F70–F79 before M-CHAT screen
Hearing disability	NPR	ICD-10: H90–91 before M-CHAT screen
Motor	NPR	ICD 10: F82, G80 before M-CHAT screen
Vision	NPR	ICD-10: H54 before M-CHAT screen
Other developmental disorders		
ADHD	NPR or PDR	ICD-10: F90 or filled prescription of ATC N06BA04 (methylphenidate), N06BA09 (atomoxetine), N06BA12 (lisdexamfetamine), C02AC02 (guanfacin), N06BA02 (dexamfetamine) during follow-up
ID	NPR	ICD-10: F70–F79 during follow-up
Speech and language	NPR	ICD-10: F80 during follow-up
Motor function	NPR	ICD-10: F82 during follow-up
Other or unspecified disorders of psychological development	NPR	ICD-10: F88 and/or F89 during follow-up
BPD	SNQ	As reported by NICU
HIE	SNQ	As reported by NICU
IVH	SNQ	As reported by NICU
NEC	SNQ	As reported by NICU
PDA	SNQ	As reported by NICU
ROP	SNQ	As reported by NICU
SGA	SNQ	As reported by NICU
Scandinavian language spoken at home	MBR	Birth weight more than 3 standard deviations below the mean for gestational age, based on sex-specific Swedish reference curves by Marsál et al.
Parental educational level	LISA	Highest maternal and paternal educational level at child birth
Date of first emigration from Sweden	Total population register	
Date of death	Total population register	

© 2026 Lassebro B et al. *JAMA Network Open*.

Abbreviations: ASD, autism spectrum disorder; NPR, National Patient Register; ICD, international statistical classification of diseases and related health problems; ADHD, attention deficit hyperactivity disorder; PDR, Prescribed Drug Register; ID, intellectual disability; BPD, bronchopulmonary dysplasia; SNQ, Swedish Neonatal Quality Register; HIE, hypoxic ischemic encephalopathy; IVH, intraventricular hemorrhage; NEC, necrotizing enterocolitis; PDA, Patent Ductus Arteriosus; ROP, Retinopathy of Prematurity; SGA, small for gestational age

© 2026 Lassebro B et al. *JAMA Network Open*.

eTable 2. Participant Characteristics by Neonatal High-Risk Group

	EPT	SGA (<-3SD)	Morphological brain damage	Neonatal encephalopathy	Other severe morbidity
Number of children (%)	870 (39.9%)	416 (19.1%)	143 (6.6%)	419 (19.2%)	330 (15.2%)
Sex Male	478 (54.9)	220 (52.9)	86 (60.1)	229 (54.7)	197 (59.7)
Female	392 (45.1)	196 (47.1)	57 (39.9)	190 (45.3)	133 (40.3)
Birthweight (g)	800 (507–1175)	1382 (737–2160)	3241 (1211–4525)	2945 (1170–4546)	3030 (1036–4430)
Small for gestational age <3SD	70 (8.1)	416 (100.0)	3 (2.1)	6 (1.5)	13 (4.1)
Gestational_age					
22-23 weeks	94 (10.8)	0 (0.0)	0 (0.0)	0 (0.0)	0 (0.0)
24-27 weeks	776 (89.2)	0 (0.0)	0 (0.0)	0 (0.0)	0 (0.0)
28-32 weeks	0 (0.0)	155 (37.3)	32 (22.4)	148 (35.3)	74 (22.4)
33-36 weeks	0 (0.0)	148 (35.6)	18 (12.6)	28 (6.7)	62 (18.8)
37-42 weeks	0 (0.0)	113 (27.2)	93 (65.0)	243 (58.0)	194 (58.8)
Bronchopulmonary Dysplasia	634 (72.9)	43 (10.3)	7 (4.9)	13 (3.1)	30 (9.1)
Necrotizing Enterocolitis	74 (8.5)	7 (1.7)	3 (2.1)	3 (0.7)	13 (3.9)
Persistent Ductus Arteriosus	532 (61.1)	13 (3.1)	3 (2.1)	20 (4.8)	18 (5.5)
IVH grade 3-4	69 (7.9)	0 (0.0)	13 (9.1)	0 (0.0)	2 (0.6)
Retinopathy of prematurity grade 3-5	228 (26.2)	1 (0.2)	1 (0.7)	0 (0.0)	1 (0.3)
HIE grade 2-3	0 (0.0)	0 (0.0)	12 (8.4)	163 (38.9)	18 (5.5)
Congenital abnormalities	467 (53.7)	137 (32.9)	42 (29.4)	123 (29.4)	109 (33.0)
Mother_education					
<=9 years	101 (11.6)	49 (11.8)	20 (14.0)	39 (9.3)	37 (11.2)
10-12 years	305 (35.1)	112 (26.9)	55 (38.5)	131 (31.3)	111 (33.6)
>12 years	446 (51.3)	243 (58.4)	64 (44.8)	241 (57.5)	176 (53.3)
Missing	18 (2.1)	12 (2.9)	4 (2.8)	8 (1.9)	6 (1.8)

© 2026 Lassebro B et al. *JAMA Network Open*.

	EPT	SGA (<-3SD)	Morphological brain damage	Neonatal encephalopathy	Other severe morbidity
Father_education					
<=9 years	125 (14.4)	54 (13.0)	20 (14.0)	46 (11.0)	39 (11.8)
10-12 years	352 (40.5)	149 (35.8)	73 (51.0)	177 (42.2)	142 (43.0)
>12 years	345 (39.7)	178 (42.8)	45 (31.5)	176 (42.0)	135 (40.9)
Missing	48 (5.5)	35 (8.4)	5 (3.5)	20 (4.8)	14 (4.2)
Maternal_age					
<20	7 (0.8)	4 (1.0)	0 (0.0)	3 (0.7)	1 (0.3)
20-29	317 (36.4)	150 (36.1)	62 (43.4)	160 (38.2)	118 (35.8)
30-39	489 (56.2)	235 (56.5)	72 (50.3)	220 (52.5)	187 (56.7)
>=40	57 (6.6)	27 (6.5)	9 (6.3)	36 (8.6)	24 (7.3)
Language_at_home					
Scandinavian	487 (56.0)	243 (58.4)	106 (74.1)	273 (65.2)	239 (72.4)
Non-Scandinavian	213 (24.5)	95 (22.8)	30 (21.0)	89 (21.2)	76 (23.0)
Missing	170 (19.5)	78 (18.8)	7 (4.9)	57 (13.6)	15 (4.5)
Year_of_birth					
2013-2015	319 (36.7)	124 (29.8)	28 (19.6)	180 (43.0)	87 (26.4)
2016-2017	288 (33.1)	131 (31.5)	52 (36.4)	138 (32.9)	111 (33.6)
2018-2019	263 (30.2)	161 (38.7)	63 (44.1)	101 (24.1)	132 (40.0)
Age at assessment (months)	26.0 (23.0–30.0)	26.0 (23.0–29.0)	26.0 (23.0–30.0)	26.0 (23.0–29.0)	26.0 (24.0–30.0)

Values are n (%) or median (5% and 95% percentiles). Abbreviations: EPT, extremely preterm (<28 gestational weeks); SGA, small for gestational age; IVH, intraventricular hemorrhage; HIE, hypoxic ischemic encephalopathy

[a, b] 40 (1.8%) were missing data on birthweight and SGA

[c] 112 (5.1%) were missing data on age at assessment.

© 2026 Lassebro B et al. *JAMA Network Open*.

eTable 3. Performance of the Modified Checklist for Autism in Toddlers for a Diagnosis of Childhood Autism, ASD+ID, and ASD+ADHD[a]

	Diagnosis (n [%])	Sensitivity (95% CI)	Specificity (95% CI)	PPV (95% CI)	NPV (95% CI)
**Childhood autism (ICD 10 code F84.0)**	128 (5.9)	64.7 (56.5-72.3)	91.2 (90.1-92.4)	31.4 (26.0-37.1)	97.7 (97.0-98.3)
M-CHAT positive	83 (31.6)				
M-CHAT negative	45 (2.3)				
**ASD+ID**	42 (1.9)	71.1 (57.7–83.5)	89.1 (87.8–90.5)	11.4 (7.7–15.4)	99.4 (99.0–99.7)
M-CHAT positive	30 (11.4)				
M-CHAT negative	12 (0.6)				
**ASD+ADHD**	30 (1.4)	29.6 (13.5–48.3)	88.2 (86.9–89.5)	3.3 (1.4–5.9)	98.9 (98.4–99.3)
M-CHAT positive	9 (3.4)				
M-CHAT negative	21 (1.1)				

Abbreviations: M-CHAT, Modified Checklist for Autism in Toddlers; ASD, autism spectrum disorder; ID, intellectual disability; ADHD, attention deficit hyperactivity disorder; ICD, international statistical classification of diseases and related health problems; PPV, positive predictive value; NPV, negative predictive value

[a] Analyses performed on the same study population of 2178 children as in the main analysis, with 263 M-CHAT positive and 1915 M-CHAT negative children.

© 2026 Lassebro B et al. *JAMA Network Open*.

eTable 4. Disabilities Present at Age 2 Years, Positive Modified Checklist for Autism in Toddlers Screens, and ASD Diagnoses at End of Follow-Up

Disability	Number of positive screens/number with disability (%)	ASD diagnoses (n (%)) (among those with disability who screened positive)
Motor	13/27 (48.1)	5 (38.5)
Vision	8/15 (53.3)	0
Hearing	15/66 (22.7)	8 (53.3)
Cognition	5/8 (62.5)	0
Neurosensory	35/107 (32.7)	12 (34.3)
Any disability	38/113 (33.6)	12 (31.6)
No disability	225/2065 (10.9)	71 (31.6)

Abbreviations: n, number of individuals; M-CHAT, Modified Checklist for Autism in Toddlers; ASD, autism spectrum disorder

© 2026 Lassebro B et al. *JAMA Network Open*.

eTable 5. Association of Having Another Language Than a Scandinavian Language Spoken at Home With Performance of Modified Checklist for Autism in Toddlers Screening to Estimate an ASD Diagnosis[c]

	ASD diagnosis (n [%])	Sensitivity (95% CI)	Specificity (95% CI)	PPV (95% CI)	NPV (95% CI)
Scandinavian mother tongue (n=1348)	66 (4.9)	58.0 (46.4–70.7)	93.3 (91.9–94.7)	30.3 (22.6–39.7)	97.7 (96.9–98.5)
M-CHAT positive[a]	38 (30.4)				
M-CHAT negative[a]	28 (2.3)				
Non-Scandinavian mother tongue (n=503)	41 (8.2)	73.3 (59.2–85.3)	86.5 (83.4–89.5)	32.2 (23.0–42.0)	97.3 (95.7–98.8)
M-CHAT positive[b]	30 (32.3)				
M-CHAT negative[b]	11 (2.7)				

Abbreviations: n, number of individuals; ASD, autism spectrum disorder; M-CHAT, Modified Checklist for Autism in Toddlers; PPV, positive predictive value; NPV, negative predictive value

[a] Number (%) of M-CHAT-positive children: 125 (9.3%). M-CHAT-negative children: 1223 (90.7%).

[b] Number (%) of M-CHAT-positive children: 93 (18.5%). M-CHAT-negative children: 410 (81.5%).

[c] 327 children were missing information on language spoken at home, and were excluded from the analysis.

© 2026 Lassebro B et al. *JAMA Network Open*.

eTable 6. Sensitivity Analyses

	M-CHAT result	ASD diagnosis (n [%])	Sensitivity (95% CI)	Specificity (95% CI)	PPV (95% CI)	NPV (95% CI)
Children born 2013-2017 (n=1458)[a]	Overall	113 (7.8)	57.5 (48.6–66.4)	91.5 (90.0–93.0)	36.3 (29.2–43.5)	96.3 (95.2–97.3)
	Positive	65 (36.3)				
	Negative	48 (3.8)				
Starting follow-up for ASD from age 3 (n=2162)[b]	Overall	117 (5.4)	59.0 (50.4–67.3)	91.2 (90.0–92.5)	27.5 (22.1–33.5)	97.5 (96.8–98.2)
	Positive	69 (27.7)				
	Negative	48 (2.5)				
Children with registered inclusion date (n=2066)[c]	Overall	127 (6.1)	62.1 (53.5–69.8)	91.3 (90.0–92.5)	31.7 (25.7–37.9)	97.4 (96.7–98.0)
	Positive	79 (31.9)				
	Negative	48 (2.6)				
Without excluding 8 children with ASD diagnosis before M-CHAT screen (n=2186) [d]	Overall	141 (6.5)	63.8 (55.8-71.4)	91.2 (90.0-92.5)	33.3 (27.8-39.2)	97.3 (96.6-98.1)
	Positive	90 (33.3)				
	Negative	51 (2.7)				

Abbreviations: n, Number of individuals; ASD, autism spectrum disorder; M-CHAT, Modified Checklist for Autism in Toddlers; PPV, positive predictive value; NPV, negative predictive value

[a] N (%) of M-CHAT-positive children: 179 (12.3%); N (%) of M-CHAT-negative children: 1279 (87.7%)

[b] N (%) of M-CHAT-positive children: 249 (11.5%); N (%) of M-CHAT-negative children: 1913 (88.5%)

[c] N (%) of M-CHAT-positive children: 248 (12.0%); N (%) of M-CHAT-negative children: 1818 (88.0%)

[d] N (%) of M-CHAT-positive children: 270 (12.4%); N (%) of M-CHAT-negative children: 1916 (87.6%)

© 2026 Lassebro B et al. *JAMA Network Open*.

eFigure 1. Flowchart of Study Population

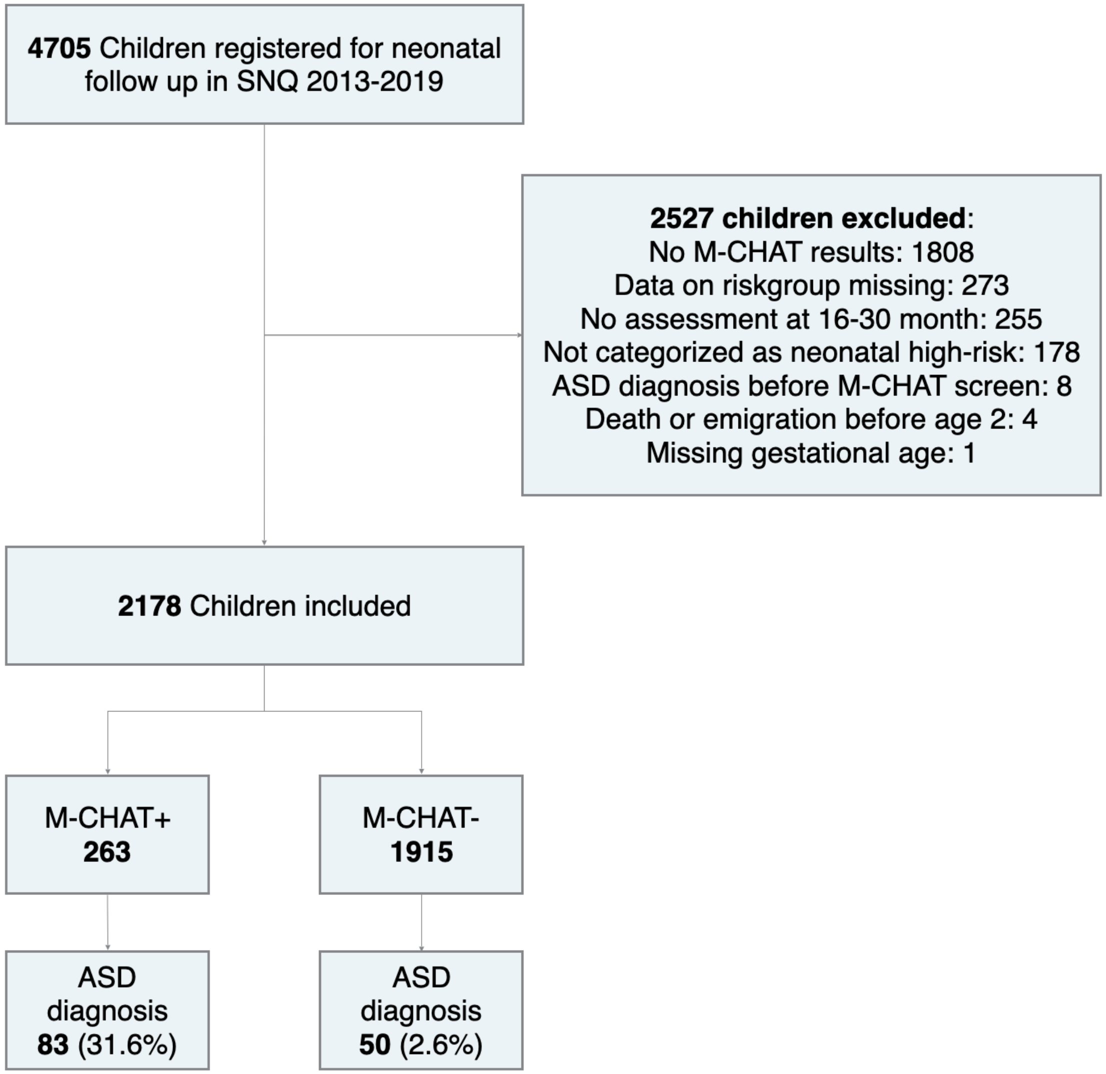

© 2026 Lassebro B et al. *JAMA Network Open*.

eFigure 2. Proportion of Other Developmental Disorders Among Individuals Who Screened Positive on the Modified Checklist for Autism in Toddlers, by ASD Status

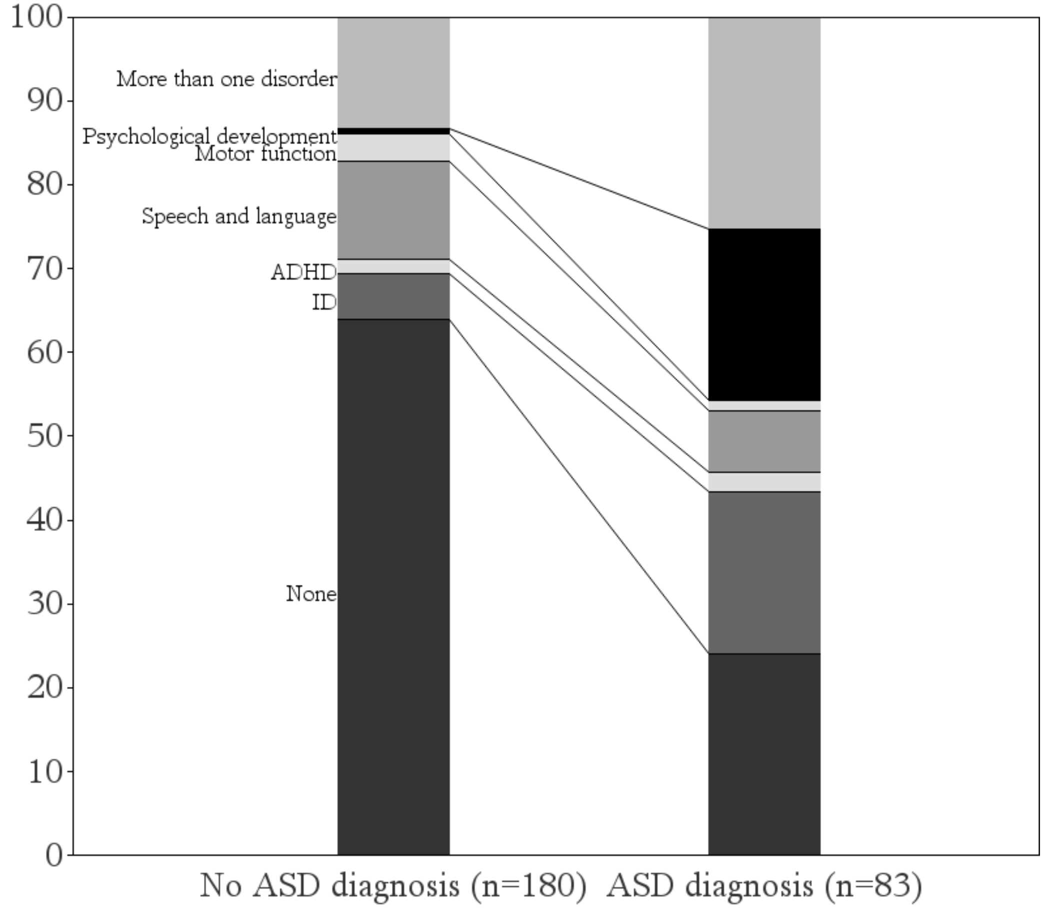

Developmental disorders were defined by diagnoses in the National Patient Register during follow-up using the following ICD-10 codes: intellectual disability (ID), ICD-10 F70-F79; attention deficit hyperactivity disorder (ADHD), ICD-10 F90; Specific developmental disorders of speech and language, ICD-10 F80; Specific developmental disorders of motor function, ICD-10 F82; Other or unspecified developmental disorders of psychological development, ICD-10 F88-F89. ASD, autism spectrum disorder.

© 2026 Lassebro B et al. *JAMA Network Open*.
