# Supplementary material for: Modified Checklist for Autism in Toddlers in a Neonatal High-Risk Population
Source: JAMA Netw Open. 2026 Mar 27;9(3):e263672. doi: 10.1001/jamanetworkopen.2026.3672 (PMC13032150; doi:10.1001/jamanetworkopen.2026.3672)
Supplement: Supplement 2. — Data Sharing Statement [file jamanetwopen-e263672-s002.pdf]

Data Sharing Statement

Data

Data available: No

Additional Information

Explanation for why data not available: Data can be shared upon request in aggregated form. Patient information, confidential.
